# Humoral Immunodeficiency with Hypotonia, Feeding Difficulties, Enteropathy, and Mild Eczema Caused by a Classical *FOXP3* Mutation

**DOI:** 10.3389/fped.2017.00037

**Published:** 2017-02-27

**Authors:** Paul Tuijnenburg, Eloy Cuadrado, Annet M. Bosch, Angelika Kindermann, Machiel H. Jansen, Marielle Alders, Ester M. M. van Leeuwen, Taco W. Kuijpers

**Affiliations:** ^1^Department of Pediatric Hematology, Immunology, Rheumatology and Infectious Diseases, Emma Children’s Hospital, Academic Medical Center (AMC), University of Amsterdam, Amsterdam, Netherlands; ^2^Department of Experimental Immunology, Academic Medical Center (AMC), University of Amsterdam, Amsterdam, Netherlands; ^3^Department of Hematopoiesis, Sanquin Research and Landsteiner Laboratory, University of Amsterdam, Amsterdam, Netherlands; ^4^Department of Metabolic Disorders, Emma Children’s Hospital, Academic Medical Center (AMC), University of Amsterdam, Amsterdam, Netherlands; ^5^Department of Pediatric Gastroenterology, Emma Children’s Hospital, Academic Medical Center (AMC), University of Amsterdam, Amsterdam, Netherlands; ^6^Department of Clinical Genetics, Academic Medical Center (AMC), University of Amsterdam, Amsterdam, Netherlands

**Keywords:** IPEX syndrome, FOXP3, Treg, autoimmunity, immunodeficiency, WES

## Abstract

We describe here the case of a boy who presented with pulmonary infections, feeding difficulties due to velopharyngeal insufficiency and gastroesophageal reflux, myopathy, and hypotonia soon after birth. Later, he was also found to have an elevated immunoglobulin (Ig) E and mild eczema and was diagnosed with inflammatory bowel disease. Further immunological screening at the age of 7 years showed low B and NK cell numbers but normal CD4^+^ and CD8^+^ T cells and notably, normal numbers of CD4^+^ regulatory T (Treg) cells. Serum IgG, IgA, and IgM were low to normal, but he had a deficient response to a pneumococcal polysaccharide vaccine and thus a humoral immunodeficiency. To our surprise, whole exome sequencing revealed a mutation in *forkhead box protein 3 (FOXP3)*, encoding an essential transcription factor for the development and function of Treg cells. This classical mutation is associated with immune dysregulation, polyendocrinopathy, enteropathy, X-linked (IPEX) syndrome. Further *in vitro* studies indeed showed defective function of Treg cells despite normal FOXP3 protein expression and nuclear localization. The boy underwent hematopoietic stem cell transplantation at 11 years of age and despite the temporary development of diabetes while on prednisone is now doing much better, IgE levels have declined, and his fatigue has improved. This case illustrates that a classical pathogenic mutation in *FOXP3* can lead to a clinical phenotype where the diagnosis of IPEX syndrome was never considered because of the lack of diabetes and the presence of only mild eczema, in addition to the normal Treg cell numbers and FOXP3 expression.

## Summary

The constellation of early onset diabetes, infections, eczema, and elevated immunoglobulin (Ig) E levels with persistent and often severe diarrhea often results in the consideration of immune dysregulation, polyendocrinopathy, enteropathy, X-linked (IPEX) or IPEX-like syndromes. In general, we learn from whole exome and genome sequencing that the spectrum of circumscribed syndromes is much broader than was previously appreciated, as also illustrated by a mild presentation of a classical mutation in the *forkhead box protein 3* (*FOXP3*) gene c.1010G>A; p.(Arg337Gln) (reference sequence NM014009.3) believed to be highly penetrant and disease causative.

## Introduction

A boy born at term of Caucasian non-consanguineous parents presented with respiratory insufficiency immediately after birth due to tracheomalacia and a diaphragmatic paresis. A diaphragmatic plication was performed at the second month after birth. The patient suffered from severe pulmonary infections necessitating respiratory support repeatedly in the first years of life. Feeding difficulties started shortly after birth due to velopharyngeal insufficiency and gastroesophageal reflux and led to the placement of a percutaneous endoscopic gastrostomy tube in the first year after birth and veloplastic surgery at the age of 5 years. There was a persistence of loose stools, and an allergy was suspected. With the differential diagnosis of cow’s milk allergy, a hydrolysate was started (Neocate). Due to bloody diarrhea, a combined gastro-duodenoscopy and colonoscopy was performed when the boy was 3 years old. Histopathology showed macroscopic colitis and microscopic crypt abscesses and active chronic inflammation in the colon, duodenum, stomach, and esophagus. Therefore, inflammatory bowel disease was diagnosed. Immunosuppressive medication (including prednisolone) was started which improved his stools and clinical well-being. In the years that followed, the symptoms of diarrhea were under control by azathioprine with occasional flares. Attempts to reduce azathioprine dose led to an immediate increase in diarrhea. However, despite immunosuppressive medication and surgical corrective interventions, the feeding difficulties persisted and pneumonias recurred. From the first day of life, there was a severe hypotonia and proximal muscle weakness causing a delayed motor development. A muscle biopsy suggested a mild deficiency of complex 1 of the mitochondrial respiratory chain, but this was never supported by genetic testing.

At the age of 7 years, the boy was sent to our clinical immunology unit because of the recurrent infections and allergy with an elevated IgE of 2,275 kU/ml in the presence of a normal white blood cell count and Ig spectrum (Table 1). We met a cooperative boy with a normal height, weight and head circumference, normal heart rate, respiration and saturation, and blood pressure. He had a slightly flaccid face and was sitting in a wheel chair, yet able to walk for a small distance, and he had generally low muscle tone, muscular weakness, and Gower’s sign upon physical examination. We found normal eye movement, vision, and hearing and normal chewing, swallowing, and tongue movements. No signs of neuropathy, no ataxia, dysarthria, or speech impairment were found. Psychomotor development was normal otherwise, although he had low yet symmetrical reflexes.

**Table 1:** **Hematology and immunology parameters in the index case at presentation**.

Laboratory results	Patient (7 years of age)	Normal range (5–12 years)
**Hematology**		
• Hemoglobin (mmol/l)	7.0	6.0–9.0
• Platelets (10^6^/ml)	220	150–350
• White blood cells (10^6^/ml)	7.4	4–16
• Neutrophils (10^6^/ml)	3.2	1.5–8.0
• Eosinophils (10^6^/ml)	0.6	<0.5
• Monocytes (10^6^/ml)	1.2	0.2–1.0
• Lymphocytes (total, 10^6^/ml)	2.4	1.5–5.0
**Immunology, humoral**		
• IgG (g/l)	6.8	6.0–10.3
• IgA (g/l)	0.26	0.3–1.7
• IgM (g/l)	0.27	0.2–0.75
• IgE (kU/l)	2,275	<100
**Vaccine response^a^**		
• Anti-PPS23 (ratio of 23 serotypes)	1.28	>2.0
• Anti-Tet-Toxoid (ratio)	27.9	>2.0
**Lymphocyte counts (cells/μl)**		
CD3^+^ T cells	1,424	700–3,500
• CD3^+^CD4^+^ T cells	691	300–2,100
• CD3^+^CD8^+^ T cells	745	200–1,200
CD4/CD8 ratio	0.9	0.9–3.6
CD3^+^CD4^+^CD8^+^	50	<35
CD3^−^CD16^+^CD56^+^ NK cells	20	70–1,200
CD19^+^ B cells	15	100–600

*^a^Data were taken at day 0 prevaccination and day 35 postvaccination for comparison and divided to calculate a ratio. The ratio should be above 2.0, taking into account the concentration of antipneumococcal antibodies or antitetanus toxoid antibodies at start (when compared to immunized controls)*.

*Ig, immunoglobulin*.

Apart from a clear periodontitis, some pulmonary fine crackles at the end-inspiratory inhalation, and mild hepatomegaly, no other remarkable findings could be found, in particular, no splenomegaly or lymphadenopathy. The boy had a dry skin without obvious eczema, also not around the PEG insertion site for enteral feeding. Apart from 350 ml daily enteral tube feeding (Nutrini Energy Multifiber), medication consisted of daily azathioprine (1.5 mg/kg body weight, bid).

### Laboratory Findings

Upon laboratory investigations, complete blood count and differentials were normal, and enzymatic liver tests, kidney, and thyroid function tests were within normal ranges. The initial immunological screening tests were abnormal with low to normal serum IgG, IgA, and IgM and very high IgE levels. Antibody response to pneumococcal polysaccharide vaccination (anti-PPS23) was deficient (Table 1). Fecal elastase levels were within the normal range excluding exocrine pancreatic dysfunction; calprotectin was elevated [ranging from normal (<50) to 1,159 μg/g of feces], and microbiological stool tests (bacterial, viral, and parasitic) were repeatedly normal.

### Immunophenotyping and Functional Tests

**Figure 1:**
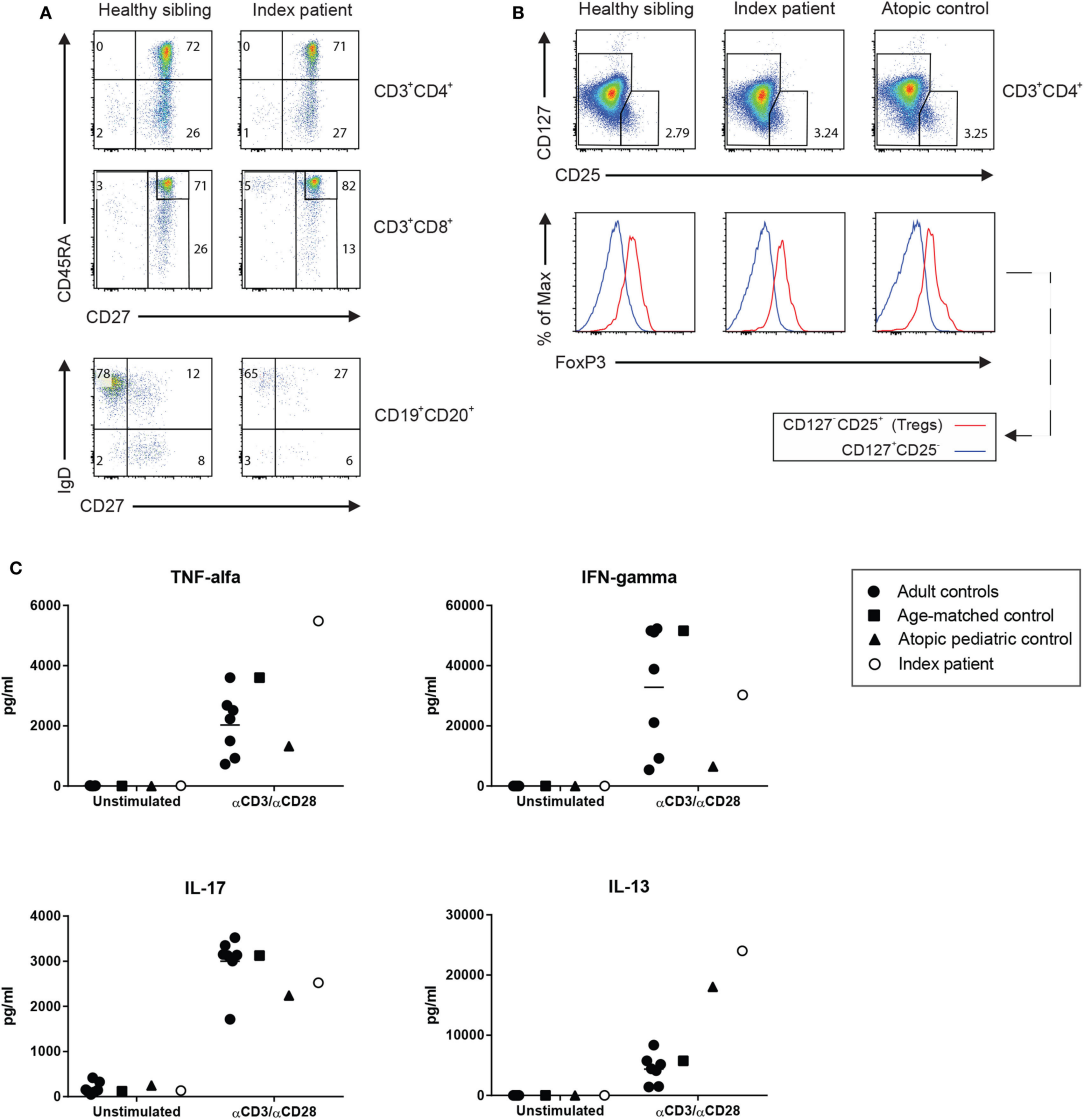
**(A)** The immunophenotyping of the T and B lymphocyte subsets of the patient was compared with the maturation of his 2-year-old brother who did not carry the mutation. **(B)** Standard screening of CD4^+^ regulatory T cells using CD127 and CD25 immunostaining (upper panel) was further supported by the assessment of a normal expression of intracellular forkhead box protein 3 (lower panel). **(C)** Function of the patient’s helper cell differentiation was tested by measuring the cytokines for the Th1 (IFNγ, TNFα), Th2 (IL-13), and Th17 (IL-17) in the supernatant of anti-CD3/anti-CD28-stimulated T cells as determined by Luminex (eBioscience, San Diego, CA, USA).

Lymphocyte enumeration was routinely done by flow cytometry and showed low numbers of B and NK cells with normal T cell numbers but a low CD4/CD8 ratio and increased CD4^+^CD8^+^ double-positive (DP) T cells (Table 1). The percentage of naïve and switched memory B cells was within age-specific reference ranges. CD4^+^ and CD8^+^ T cell differentiation into memory/effector memory cells was normal (Figure 1A). The percentage of CD4^+^ regulatory T (Treg) cells determined by CD25 and CD127 was found to be unremarkable, and the intracellular FOXP3 expression was comparable to healthy controls (Figure 1B). Lymphocyte activation using T cell stimuli such as anti-CD3/anti-CD28 or IL-15 induced adequate proliferative responses of CD4^+^ and CD8^+^ T cells as assessed by carboxyfluorescein succinimidyl ester dilution (data not shown), although the cytokine release into the supernatant was considered high to normal, in particular for the Th2 signature cytokine IL-13 (Figure 1C).

### Genetics

**Figure 2:**
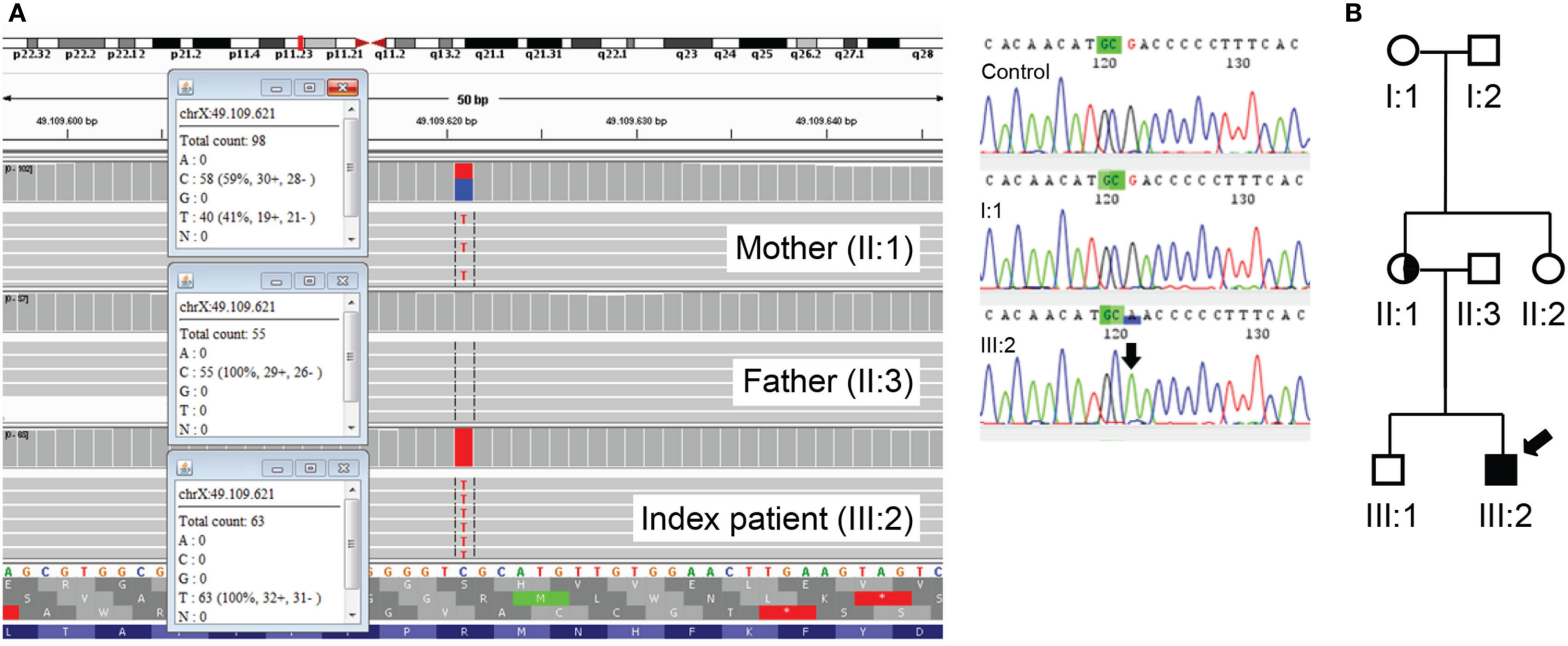
**(A)** Mutation c.1010G>A; p.(Arg337Gln) in the *forkhead box protein 3 (FOXP3)* gene identified using whole exome sequencing (gene on reverse strand). The index patient is hemizygous, and the mother is a heterozygous carrier (left panel). Mutation c.1010G>A; p.(Arg337Gln) in the *FOXP3* gene is not present in the grandmother, I:1 (right panel). **(B)** The cosegregation study in the family showing that the mutation had occurred as a *de novo* change in the mother of the patient. Arrow indicates index patient.

Whole exome sequencing (WES) was performed in a trio diagnostic approach (patient and both parents). In short, libraries were prepared using the Kapa HTP kit (Illumina, San Diego, CA, USA), and capture was performed using the SeqCap EZ Exome v3.0 (Roche Nimblegen Madison, WI, USA). Sequencing was done on an Illumina HiSeq2500 HTv4 (Illumina, San Diego, CA, USA) with paired-end 125-bp reads. Read alignment to hg19 and variant calling were done with a pipeline based on BWA-MEM0.7 and GATK 3.3. Variant annotation and prioritizing were done using Cartagenia NGS Bench (Cartagenia Inc., Cambridge, MA, USA). This revealed a hemizygous mutation c.1010G>A; p.(Arg337Gln) in the *FOXP3* gene (NM_014009.3) in the patient. His mother was found heterozygous for the mutation (Figure 2A). This mutation has been described as causal for IPEX syndrome (1–4) and is not present in the Exac database (http://exac.broadinstitute.org). Subsequent cosegregation showed that the mutation occurred *de novo* in the mother (Figure 2B).

### Treg Cell Function

**Figure 3:**
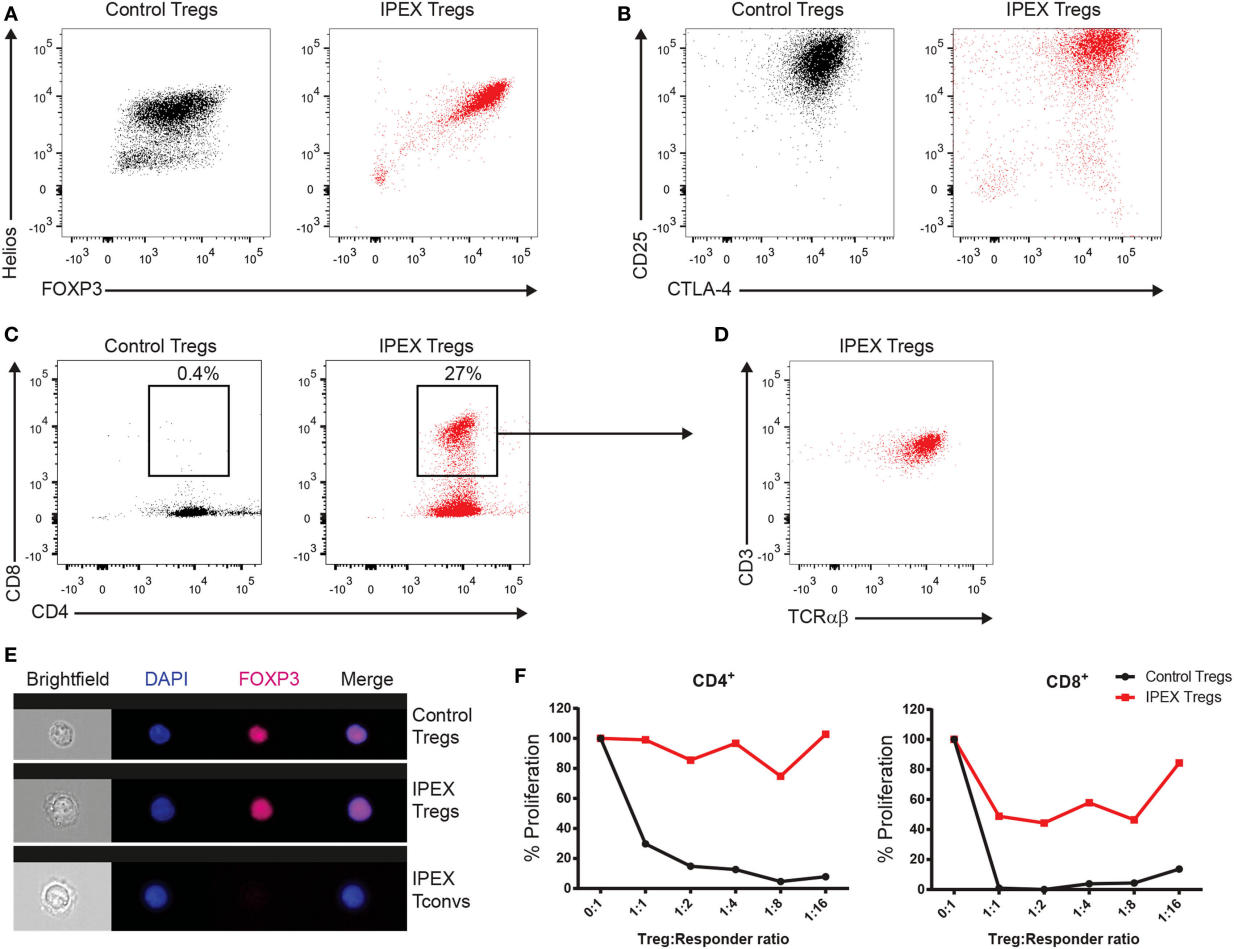
**(A)** FACS panels showing the intracellular staining for forkhead box protein 3 (FOXP3) and Helios in the *in vitro* expanded regulatory T (Treg) cells from a healthy donor (in black) and the immune dysregulation, polyendocrinopathy, enteropathy, X-linked (IPEX) syndrome patient (in red). **(B)** FACS plots displaying the expression of CD25 and CTLA-4. **(C)** FACS plots of CD4 and CD8 staining. Gate encloses the CD4^+^CD8^+^ double-positive (DP) cells. **(D)** Immunophenotyping for CD3 and TCRαβ of the cells in the gate in panel **(C)**. **(E)** Representative images of ImageStream analysis from Treg and conventional T cells. Nuclear localization was confirmed by colocalization of the FOXP3 staining (purple) with the nuclear DAPI signal (in blue). **(F)** Graphs representing the proliferation of responder CD4^+^ or CD8^+^ T cells gated from total allogeneic peripheral blood mononuclear cells normalized to the proliferation of the responders only condition (0:1).

Once the mutation in *FOXP3* was found in the presence of normal FOXP3 protein expression, we analyzed the Treg cells more closely by investigating their functional capacities *in vitro*. Naïve CD45^+^CD4^+^CD25^+^ Treg cells from the patient and a healthy donor were expanded upon stimulation with anti-CD3/anti-CD28. The cells were subsequently stained for the expression of the quintessential Treg cell transcription factors FOXP3 and Helios (Figure 3A) and the surface molecules CD25 and CTLA-4 (Figure 3B). In both cases, the expression of those proteins was comparable to a healthy donor. Also, the Treg cells of the patient were able to normally produce both IL-10 and TGF-β and did not produce effector cytokines such as IFNγ (data not shown). Remarkably, the patient’s Treg cells presented an elevated presence of CD4^+^CD8^+^ DP cells that were also CD3^+^TCRαβ^+^ (Figures 3C,D).

Some *FOXP3* mutations have been described to affect the nuclear localization of the protein (5); we therefore analyzed the nuclear localization of FOXP3 in the Treg cells of the patient by ImageStream (Figure 3E). Results showed a normal FOXP3 nuclear distribution comparable to that of the healthy donor. Finally, we assessed the functional capacity of the Treg cells to suppress the proliferation of CD4^+^ and CD8^+^ T cells. Undoubtedly, the Treg cells were unable to fully suppress the proliferation of neither CD4^+^ nor CD8^+^ T cells, although the Treg cells still retained a limited capacity to suppress CD8^+^ T cell proliferation (Figure 3F).

## Background

### History and Review

Immune dysregulation, polyendocrinopathy, enteropathy X-linked syndrome (OMIM304790) is an X-linked genetic disorder characterized by enteropathy, insulin-dependent type-1 autoimmune diabetes mellitus (IDDM1), and eczema. Severe enteropathy manifesting as watery or bloody diarrhea is an early feature of the disease present in all IPEX syndrome patients. Furthermore, IDDM1 is common (>80%) and is diagnosed early, often within the first 1–3 months of life (6–11). It was found in 4% of neonatal permanent diabetes among males (2). Other prominent symptoms due to profound immune dysregulation in males with IPEX syndrome include eczema/dermatitis (~65%), failure to thrive (~50%), thyroiditis (~30%), and recurrent infections (~20%). Autoimmune phenomena such as nephropathy, pneumonitis, hepatitis, vasculitis, arthritis, myositis, alopecia, and autoimmune cytopenias may be present (11, 12). The severe watery diarrhea and life-threatening infections reduce the lifespan of these patients, and they usually die in the first years of life.

In case of elevated IgE levels, the first entities to be considered are atopic disease and allergy, parasitic infestations, *Aspergillus* infection (allergic bronchopulmonary aspergillosis) in cystic fibrosis, and secondary to HIV infection. Although allergy was indicated by positive RAST tests against milk protein, soja protein, casein, lactalbumin, lactoglobulin, and chicken protein, all other causes for the increased IgE levels were excluded in our patient. Primary immunodeficiency disorders (PIDs) such as hyper-IgE syndrome, Wiskott–Aldrich syndrome, leaky severe combined immunodeficiency/Omenn syndrome, autoimmune polyendocrinopathy-candidiasis-ectodermal dystrophy, or other rare disorders such as IPEX-like disease (for instance due to *FOXP3, WASP, CD25, RAG1/2, AIRE*, carbohydrate defect in glycosylation-variant *PGM3* mutations, or *STAT3* or *STAT1* gain-of-function mutations) were considered (13, 14).

In patients with PIDs, a breakdown of self-tolerance mechanisms or immune dysregulation often exists, with high incidence of autoimmunity and autoinflammation as a consequence. In the patients with IPEX syndrome, the lack of FOXP3 in lymphoid tissues (thymus, spleen, and lymph nodes), as encoded by the *FOXP3* gene at Xp11.23-Xq13.3, leads to impaired development and suppressed function of CD4^+^CD25^+^ Treg cells. FOXP3 functions as the master transcription factor for the development of Treg cells, in both human and mice, together with STAT5a, and the high-affinity IL-2 receptor CD25. Beyond its role in Treg cell differentiation, continuous FOXP3 expression is also required in mature Treg cells for their suppressive function and the full manifestation of the aforementioned key features of these Treg cells. The formation of Treg cell identity, characterized by its unique transcriptional program, requires the activation of additional genes. Indeed, enforced expression of the genes encoding IKAROS family zinc finger 4 (Eos), interferon regulatory factor 4, SATB homeobox 1, lymphoid enhancer-binding factor 1, or GATA-binding protein 3 cooperate with FOXP3 to activate the expression of most, but still not all, of the Treg cell signature genes (15).

### Immune Dysregulation and Autoimmunity

The protein FOXP3 has four functional domains: an *N*-terminal proline-rich repressor domain, a zinc finger domain, a leucine zipper domain, and a *C*-terminal DNA-binding forkhead/winged helix domain. Mutations that affect any of these key functional domains may alter or abrogate the ability of the transcription factor to regulate gene expression in Treg cells. Mature naïve B cells from IPEX patients often express autoreactive antibodies, suggesting an important role for Treg cells in maintaining peripheral B cell tolerance and B cell anergy (16–18).

The B cell numbers in our patients were rather low and showed an impaired pneumococcal polysaccharide vaccination response. Remarkably, there were no autoreactive antibodies detectable in our patient. Although anti-GAD or anti-islet cell antibodies were absent, a systematic investigation of all possible organ-specific autoantibodies was not performed. There was no evidence of autoimmune cytopenia. Also autoantibodies against nuclear antigens (ANA, ENA), neutrophil cytoplasmic proteins (ANCA-IFT, MPO, and PR3), and cardiolipin were all repeatedly negative (data not shown). Still, the complete lack of autoantibodies cannot be confirmed definitely.

### Classical Mutation but Variable Penetrance

The pathogenicity of substitutions at position 337 (R337P and R337Q), similar to the one found in our patient, was reported previously in IPEX syndrome patients (1–4). In normal FOXP3, the side chain of arginine at position 337 is predicted to make close contact with the DNA backbone. The presence of a strong basic residue at this position is conserved in all forkhead transcription factors and contributes to DNA binding through both hydrogen bonding and electrostatic interactions. The R337Q substitution destroys this close contact with the DNA backbone and is predicted to cause a significant loss of positive charge at the DNA-binding surface of FOXP3, previously suggested to result in a significant loss of DNA-binding affinity which leads to severe IPEX syndrome (2). Nonetheless our case is noticeably moderately affected—even though he is carrying the same deleterious mutation at the arginine position 337.

## Discussion

### Diagnosis

The immunological studies in our patient led to the identification of a humoral immunodeficiency with low B cell counts. T cell numbers including Treg cells and their FOXP3 expression seemed normal. Only mild eczema was noted, and the use of local ointments other than cetomacrogol and vaseline cremors was not required. Past muscle biopsy and blood tests for pathological, biochemical, and metabolic causes had remained uninformative, and sequencing of his mitochondrial DNA did not result in an explanation for his muscular weakness. Yet, the diagnosis of inflammatory bowel disease at the age of 3 years is unusual and increased the chances of the cause being a monogenic disorder. A previous comparative genomic hybridization (CGH) array did not yield any relevant finding, and we did not diagnose the patient until WES was performed, which to our surprise resulted in the identification of a classical mutation in the *FOXP3* gene which fits the diagnosis of IPEX syndrome. Even when the patient would not have presented with the myopathy, the absence of diabetes as well as the normal Treg cell number and FOXP3 expression refrained us from considering Sanger sequencing *FOXP3* in first instance.

There are three evolutionally conserved non-coding sequences in the *FOXP3* gene, one located at the 5′UTR of *FOXP3* and identified as a Treg cell-specific demethylated region (19). This demethylated CpG island supports stable expression of FOXP3. This has been found to be constantly demethylated exclusively in Treg but not in T-effector cells, where it is fully methylated, which can be used to differentiate peripheral Treg from T-effector cells (which are CD25 and FOXP3 positive when activated during chronic inflammation) (17). In addition to its important role in both thymic and peripheral Treg cell differentiation, IL-2 signaling also promotes stable FOXP3 expression in mature Treg cells. Although the Treg cell number was normal by standard phenotyping assays including IL-2Rα (CD25) and FOXP3 expression, their function was clearly impaired. The patient’s Treg cells were completely unable to suppress CD4^+^ T cell proliferation. Only a very limited capacity to suppress CD8^+^ T cells was spared in the Treg cells of the patient, very possibly linked to the yet functional expression of CD25, which has been shown to be the major suppressive mechanism against CD8^+^ T cells (20). Also striking was the elevated presence of CD4^+^CD8^+^ DP cells derived from naïve Treg cells. These cells have been described in several autoimmune diseases, but their regulatory or inflammatory phenotype is still under debate (21).

### Treatment

Initial treatment of IPEX patients consists of immunosuppressive medication which is often a combination of different drugs, including corticosteroids and calcineurin inhibitors. The only curative treatment consists of hematopoietic stem cell transplantation (HSCT). HSCT was performed at the age of 11 years with a matched unrelated donor (10/10 HLA class I/II), and after a short period of diabetes while being on prednisone because of mild graft-versus-host disease, he recovered fully and did not need any insulin afterward.

Post-HSCT, his IgE levels normalized within 6 months. Also his fatigue and daily activity improved which may be due to a combination of less pulmonary inflammation and myositis, a symptom that has been reported in IPEX syndrome before (1, 12). We cannot exclude that there are other defects not identified by CGH and WES that may explain part of his birth defects.

## Concluding Remarks

1. Immune dysregulation, polyendocrinopathy, enteropathy, X-linked syndrome is a very rare disease. Patients with a mild presentation are likely to be underreported, and the true incidence of the disease might actually be higher than is currently perceived, although in the era of WES and WGS, the number of reported cases have not steeply risen yet.
2. Identical mutations in IPEX syndrome may vary from very severe to less penetrant disease, underpinning the lack of a clear-cut genotype–phenotype relationship.
3. The number of CD4^+^CD25^+^FOXP3^+^ Treg cell may be completely normal in the presence of a genetically proven “classical” IPEX mutation.
4. Immune dysregulation, polyendocrinopathy, enteropathy, X-linked syndrome may present with odd features, such as myopathy, B cell impairments, and CD4^+^CD8^+^ DP T cells.
5. Hematopoietic stem cell transplantation is a curative procedure in IPEX syndrome.

## Ethics Statement

This case report was approved by the Medical Ethical Committee of the Academic Medical Center in Amsterdam. All subjects gave written informed consent in accordance with the Declaration of Helsinki.

## Author Contributions

PT wrote the article under the supervision of EL and TK who conceptualized the paper; PT performed and analyzed some of the immunological tests together with MJ and EL. EC performed Treg function tests. TK is treating the patient together with AB and AK; MA analyzed the WES results.

## Conflict of Interest Statement

The authors declare that the research was conducted in the absence of any commercial or financial relationships that could be construed as a potential conflict of interest.
